# CTL-mediated immunotherapy can suppress SHIV rebound in ART-free macaques

**DOI:** 10.1038/s41467-019-09725-6

**Published:** 2019-05-21

**Authors:** Jin Fan, Hua Liang, Xiaolin Ji, Shuo Wang, Jing Xue, Dan Li, Hong Peng, Chuan Qin, Cassian Yee, Yiming Shao

**Affiliations:** 1 Department of Microbiology & Infectious Disease Center, School of Basic Medical Sciences, Peking University Health Science Center, Beijing, China; 2 State Key Laboratory of Infectious Disease Prevention and Control, National Center for AIDS/STD Control and Prevention, Chinese Center for Disease Control and Prevention, Collaborative Innovation Center for Diagnosis and Treatment of Infectious Diseases, Beijing, China; 3 Key Laboratory of Human Disease Comparative Medicine, Chinese Ministry of Health, Beijing Key Laboratory for Animal Models of Emerging and Remerging Infectious Diseases, Institute of Laboratory Animal Science, Chinese Academy of Medical Sciences and Comparative Medicine Center, Peking Union Medical College, Beijing, China; 4 Department of Melanoma Medical Oncology, Department of Immunology, UT MD Anderson Cancer Center, Houston, USA

**Keywords:** HIV infections, HIV infections, HIV infections, Cytotoxic T cells, Cytotoxic T cells

## Abstract

A major barrier to human immunodeficiency virus (HIV) cure is the existence of viral reservoirs that lead to viral rebound following discontinuation of antiretroviral therapy (ART). We postulate that enhancing cytotoxic T lymphocytes (CTL) targeting conserved envelope (Env) regions can eliminate HIV infected cells in latency. Here, we evaluate the use of adoptively transferred HIV vaccine-induced subtype C Env-specific CTLs in a macaque subtype B simian-human immunodeficiency virus (SHIV) model to determine whether plasma viremia can be controlled after ART interruption. We demonstrate that adoptive cellular therapy (ACT) using autologous Env-specific T cells augmented by therapeutic vaccination can suppress ART-free viral rebound in the SHIV model. Furthermore, phenotypic and functional characterization of adoptively transferred cells in ACT-responsive and nonresponsive animals support a critical role for cross-reactive central memory T cells in viremia control. Our study offers an approach to potentiate immunological suppression of HIV in the absence of antiviral drugs.

## Abstract (web summary)

Viral rebound following discontinuation of antiretroviral therapy (ART) is a major obstacle for HIV cure. Here, the authors show that adoptive cellular therapy using autologous Env-specific T cells augmented by therapeutic vaccination can control viral rebound after ART interruption in a SHIV macaque model.

## Introduction

HIV infection continues to pose a critical risk to health in many countries^1^. Despite the unquestionable success of ART, the treatment itself is unable to completely eradicate the infection, which persists in latent reservoir cells^2^. Rapid rebound in viremia and re-establishment of the HIV reservoir occur within 2–3 weeks following discontinuation of ART^3^. Given the challenges of providing lifelong therapy to a global population of almost 40 million HIV infected people, there are significant costs to providing ART to the less than 60% of HIV infected people currently being treated and the greater than 40% yet to be treated, in order to achieve the WHO/UNAIDS goal of 90-90-90 in 2020^4^. Therefore, novel immunotherapeutic strategies that can induce long-term immune-mediated control of HIV replication in the absence of ART are highly desirable, which is the ultimate goal of HIV functional cure. Recently, Borducchi et al^5^ showed that therapeutic vaccination with Ad26/MVA could expand the breadth of SIV-specific cellular immune responses in virologically suppressed macaques, improve virologic control and delay viral rebound after ART interruption. Here we test in rhesus macaques a strategy for HIV functional cure: HIV vaccine immunization combined with antiviral treatment and suppress viral rebound with CTL immunotherapy after ART interruption. We find that vaccination in ART-treated SHIV-SF162P3 -infected macaques prior to peripheral blood harvest expands and facilitates in vitro enrichment of low frequency Env-specific T cells. We then show that adoptive transfer of augmented Env-specific T cells with a central memory phenotype leads to virological suppression following drug discontinuation and viral rebound. Our study shows, in the setting of viral rebound in an ART interruption model, that therapeutic vaccination in combination with CTL immunotherapy can be a promising approach for virologic suppression.

## Results

### Animal model and experiment design

**Fig. 1:**
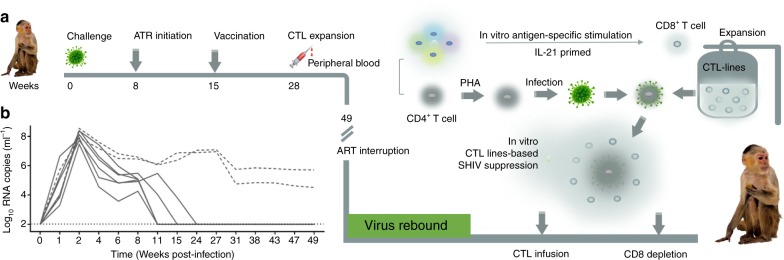
Experiment design and major procedures intervention. **a** Experiment design. **b** Viral RNA in SHIV-SF162P3 infected macaques before ART interruption (limit of detection, 2 Log RNA copies per ml). Dotted lines indicate the macaques without viral control during ART

Eight Chinese-origin rhesus macaques were inoculated intrarectally with 3 × 10^6^ TCID_50_ (50% tissue culture infective dose) SHIV-SF162P3 on day 0 (Fig. 1a). Beginning on week 8 after infection, each macaque received ART (3TC/50 mg/kg/once daily + LPV/r/15 mg/kg/twice daily). The macaques had a median plasma SHIV RNA level of 5.46 Log copies per ml (range 4.25–6.58 Log copies per ml) on the day of ART initiation. SHIV RNA levels were controlled in the majority of macaques (Animal 1-4 and 7-8, Fig. 1b, solid lines) except in two (Animal 5 and 6, Fig. 1b, dotted lines). We sequenced the SHIV viruses from the two uncontrolled viremia macaques and several controlled macaques as control at various time points of the study. The drug resistance mutation assay was performed for both RT (3TC) and PR (LPV/r) regions of the SHIV virus. The M184V drug resistance mutation to 3TC was found only in animal 5 and 6, after 3 weeks of ART. The M184V mutation lead to high resistance to 3TC. The M184V mutation persisted throughout the remainder of the study and correlated directly with elevated viral load in these two macaques (Supplementary Table 1). During ART, macaques were vaccinated by the intramuscular route with three vaccinations with DNA vaccine expressing HIV-1_CN54_-*gp140* and SIV_Mac239_-*gag* at weeks15, 19 and 22. At week 26, a booster vaccination was given by intradermal route with replication-competent recombinant Tiantan vaccinia virus expressing HIV-1_CN54_ *gag, pol* and *env -gp140* genes (rTV_gpe_) and SIV _Mac239-_*gag*. Based on previously established methods in immunized animals, peak CTL immunogenicity can be observed at 2 weeks after the rTV_gpe_ vaccine boost immunization^6^. We drew blood from the rTV immunized macaques at week 28 for T-cell generation. After interleukin-21 (IL-21) -primed^7–11^, Env-specific stimulation and isolation, the CD8^+^T-cells underwent rapid expansion (several thousand-fold expansion) resulting in 1–10 billion cells within 8 weeks for adoptive transfer to ART-free SHIV-rebound rhesus macaques (Fig. 1a).

### Kinetics of virological control

**Fig. 2:**
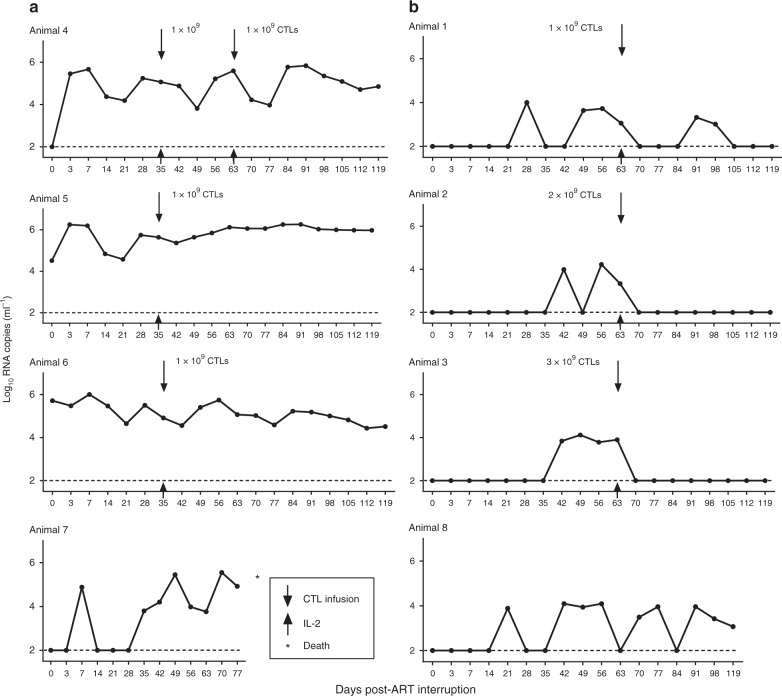
Viral RNA following ART discontinuation. Log viral RNA copies per ml are shown (limit of detection, 2 Log RNA copies per ml). **a** Macaques (*n* = 4) with high viral burden after ART interruption. **b** Macaques (*n* = 4) with low viral burden after ART interruption

Following termination of suppressive ART at 41 weeks, the macaques were split into two groups on the basis of the pattern of plasma viral load after viral rebound: (1) macaques with high viral burden (4.0–6.0 Log RNA copies per ml, *n* = 4, Fig. 2a) and (2) macaques with low viral burden (3.0–4.0 Log RNA copies per ml, *n* = 4, Fig. 2b). We performed a single infusion of 1 × 10^9^ autologous Env-specific CTLs in 3 macaques with a high viral burden (4.25–6.58 Log copies per ml, Fig. 2a, Animals 4, 5, 6) after ART interruption. One macaque (Animal-4) with early viral rebound (after 3 days) received two separate CTL infusions which each resulted in 2 Log reduction of plasma viral load within 2 weeks. Two macaques (Animals 5 and 6) lacking viral control during ART, experienced both a transient small decrease in plasma viral load. To confirm the effect of CTL intervention, we performed a second CTL infusion of 1 × 10^9^ cells in Animal-4, following which, a 2 Log reduction of the plasma viral load was again observed. One macaque (Animal-7) which did not receive CTL infusion died at 11 weeks after ART interruption (Fig. 2a). Based on this first experience of CTL administration, we performed a single CTL infusion of 3 × 10^9^ cells (*n* = 1), 2 × 10^9^ cells (*n* = 1) or 1 × 10^9^ cells in the three macaques with late viral rebound (3.0–3.8 Log RNA copies per ml, Fig. 2b, Animal 1, 2, 3). All of these macaques receiving ACT demonstrated rapid suppression of relapsing viremia to undetectable levels ( < 100 SHIV RNA copies per ml) for a period of >2 months. In the macaque (Animal-1) which received the lowest total number of CTLs (1 × 10^9^ cells), the low viremia rebound observed was converted to undetectable levels accompanied by expansion of Env-specific CD8^+^T-cells (Supplementary Figure 1). The other two macaques with low viral burden (Animals 2 and 3) received higher doses (2 and 3 × 10^9^ CTLs, respectively) of infused CTLs, and exhibited long-term viremia suppression. Untreated macaque (Animal-8) had a sustained viremia (Fig. 2b). Collectively, these data indicate that CTL-mediated immunotherapy was able to effectively suppress low level viremia following ART discontinuation, with durable reduction of high levels of plasma viremia. Viremia levels in drug-resistant macaques however were not controlled with a single dose of Env-specific CTLs.

### Antiviral immune responses of host T-cell

**Fig. 3:**
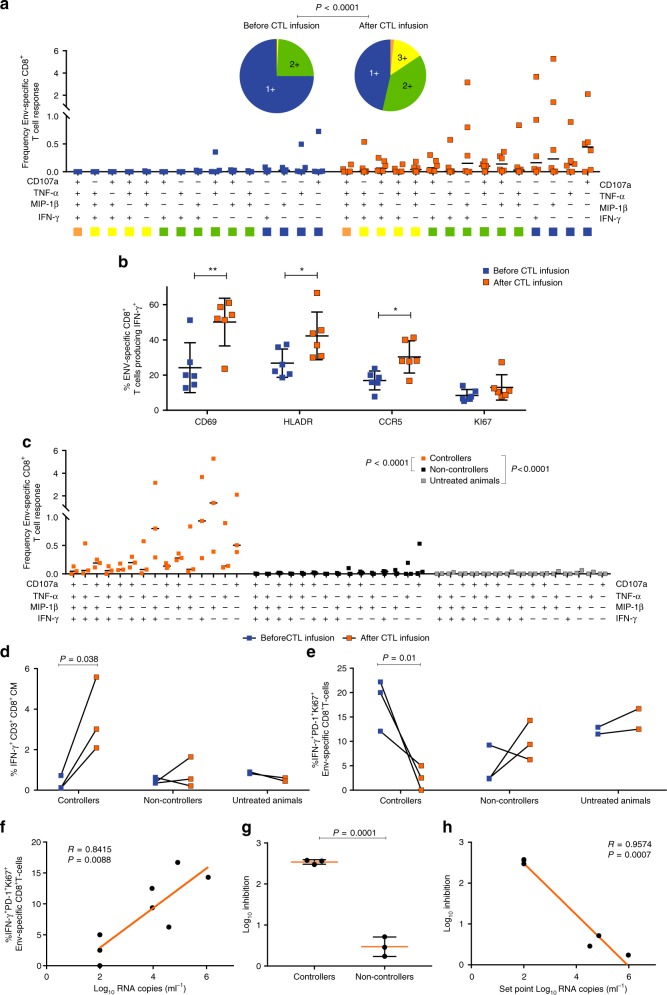
Phenotypic and functional characteristics pre- and post CTL infusion. **a** Functional profiles of CD8^+^T-cells pre- and post- CTL were evaluated by peptide antigen-specific responses and shown as the frequencies of Env-specific CD8^+^T-cells producing CD107a, IFN-γ, MIP-1β and TNF-α (*p* < 0.0001 using Wilcoxon signed-rank test, *n* = 6). **b** Activation profile of Env-specific, IFN-γ-producing CD8^+^T-cells for both pre- and post-infusion responses, based on surface expression of CD69, HLADR, CCR5 and intracellular expression of Ki67 (*p < 0.05 and **p < 0.01 using unpaired Students *t* test, Mean ± s.d. are presented in all graphs, *n* = 6). **c** Env-peptide functional profiles of CD8^+^ T-cells in controllers, non-controllers and untreated animals (*p* < 0.0001 using Wilcoxon signed-rank test, *n* = 8). **d** Env-specific, IFN-γ^+^CD3^+^CD8^+^ central memory T cells pre-and post-CTL infusion among controllers, non-controllers and untreated animals (*p* = 0.038 using paired Students t-test, *n* = 8). **e** PD-1^+^Ki67^+^expression on Env-specific CD8^+^ T-cells pre- and post-CTL infusion among controllers, non-controllers and untreated animals (*p* = 0.01 using paired Students t-test, *n* = 8). **f** Env-specific CD8^+^T-cells expressing PD-1^+^Ki67^+^ were positively correlated with viral load post infusion (Spearman’s correlation coefficient (R) and P values are indicated. Note that x-axis is log_10_ scales, *n* = 8). **g** The SHIV-suppressive activity of circulating CD8^+^T-cells from controllers and non-controllers were measured on autologous activated CD4^+^T-cells infected with SHIV-162P3. SHIV suppression was reported as the Log inhibition in p27 titers in CD4^+^T-cells supernatants when autologous ex vivo Env-stimulated CD8^+^T-cells were added at a 1:1 ratio to the culture (*p* = 0.0001 using unpaired Students *t* test, Mean ± s.d. is presented, *n* = 6). **h** Relationship between SHIV-suppressive activity (% inhibition) of circulating CD8^+^T-cells and set point of viral load (Spearman’s correlation coefficient (R) and P values are indicated. Note that x- and y-axis are log_10_ scales, *n* = 6)

Antigen-specific CD8^+^T-cells responses were measured pre- and post-CTL infusion in both SHIV controllers (Animals 1–3), non-controllers (Animals 4–6) and untreated macaques (Animals 7 and 8). After CTL infusion, we observed strong poly-functionality of Env-specific CTLs responses (Fig. 3a and Supplementary Fig. 2a) as well as significantly improved functional CTL activation. Surface staining for CD69 on Env-specific CD8^+^T-cells increased from 24.22% pre-infusion to 50.15% post-infusion; HLA-DR increased from 26.78% to 42.28% and CCR5 increased from 16.93% to 30.33%, suggesting that CTL infusion induced activation of Env-specific CD8^+^T-cells (Fig. 3b and Supplementary Fig. 2b). Furthermore, we found that the SHIV controller macaques yielded Env-specific CTL with the phenotypic and functional qualities of central memory type T cells compared with non-controllers and untreated animals. After CTL infusion, controller macaques showed significantly increased fraction of Env-specific CTLs with stronger poly-functional response (Fig. 3c and Supplementary Fig. 2a), central memory phenotype expression (CD95^+^CD28^+^CCR7^+^, Fig. 3d and Supplementary Fig. 2d), and lower PD-1^+^Ki67^+^ signaling (Fig. 3e and Supplementary Fig. 2c). By contrast, non-controllers showed upregulation of PD-1^+^Ki67^+^expression that was closely correlated with their higher plasma viremia levels (Fig. 3f). Moreover, we found that controllers were associated with a remarkably strong capacity of circulating CD8^+^T-cells to suppress the SHIV replication. Antiviral activity of these T cells was significantly higher than those of non-controllers (Fig. 3g). The fact that plasma viral load was inversely correlated with antiviral activity (Fig. 3h) strengthened the claim that adoptive transfer of a polyclonal population of Env-specific CD8^+^T-cells with documented antiviral activity can potentially lead to control of plasma viremia.

### Phenotypic and functional characterization of CTL-lines

**Fig. 4:**
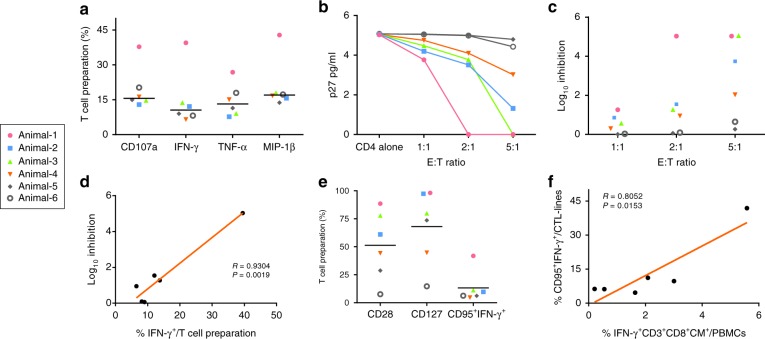
Phenotypic and functional characterization of CTL-lines. **a** In vitro specific antigen recognition of CTL-lines. Data is shown as Grand median for each group. Each symbol represented one animal (*n* = 6). **b** P27 titers in the culture supernatants of PHA-activated CD4^+^ T-cells infected in vitro with replicative SHIV-SF162P3, in the absence (CD4 alone) or presence of autologous CTL Lines (1:1, 2:1 and 5:1 of ET ratio). Each symbol represented one animal (*n* = 6). **c** The capacity of CTL-lines to suppress SHIV replication in autologous CD4^+^T-cells. The Log of p27 decreased when CD4^+^ T-cells were cocultured with CTL-lines. Each symbol represented one animal (*n* = 6). **d** Correlation between cytotoxic capacity (% inhibition) and frequencies of Env-specific IFN- γ producing (Spearman’s correlation coefficient (R) and P values are indicated. Note that y-axis is log_10_ scales, *n* = 6). **e** Memory phenotypic characterization of CTL-lines. Data is shown as Grand median for each group. Each symbol represented one animal (*n* = 6). **f** Relationship between PBMCs Env-specific, IFN-γ^+^CD3^+^CD8^+^ central memory T cells post-infusion and Env-specific memory response of CTL-lines (Spearman’s correlation coefficient (R) and *P* values are indicated, *n* = 6)

To further investigate the function of the transferred CTL-lines, we analyzed the specific antigen recognition and antiviral activity of each T-cell preparation. All CTL-lines demonstrated upregulation of CD107a and intracellular production of IFN-γ, TNF-α and MIP-1β with medians of 19.49%, 14.84%, 14.69% and 20.72%, respectively (Fig. 4a and Supplementary Fig. 2e). With a particularly robust response in Animal-1 (37.83%, 39.5%, 26.85% and 42.85%, respectively). By using an in vitro viral inhibition assay that is associated with in vivo viral control, we observed a significant, dose-dependent, suppressive effect on SHIV infected autologous CD4 target cells mediated by CTL-lines (Fig. 4b, c). We also assessed the cytotoxicity of CTL-lines against virally infected cells by assaying surface CD107a (lysosomal associated membrane protein-1, a CTL degranulation marker) expression following co-culture with SHIV infected autologous CD4^+^ T-cells (Supplementary Table 2). All CTL-lines demonstrated virus-specific responses to infected target cells by CD107a upregulation. Consistent with in vivo data, a key factor in the induction of sustained virologic suppression with ACT was Env-specificity and magnitude of effector-to-target ratio. The SHIV-suppressive capacity of CTL-lines was strongly correlated with the specific Env recognition (Fig. 4d).

Since enrichment of a central memory phenotype is associated with in vivo persistence following adoptive transfer^8,10,11^ CTL-lines were characterized for expression of central memory markers, CD28 and CD127 following stimulation with SHIV-SF162P3-infected autologous CD4^+^T-cells. The CTL-lines of controller macaques (Animals 1–3) demonstrated significantly elevated early central memory markers (CD28^hi^ and CD127^hi^, Fig. 4e and Supplementary Fig. 2f); a statistically significant correlation was observed between the frequency of circulating Env-specific central memory response in vivo (as a fraction of circulating PBMCs) and the memory response of CTL-lines in vitro (Fig. 4f). These data further support the conclusion that transferred CTLs facilitate Env-specific CD8^+^T-cell responses in SHIV controller macaques in an antigen-dependent manner.

### Env-specific response and the correlates of viremia control

**Fig. 5:**
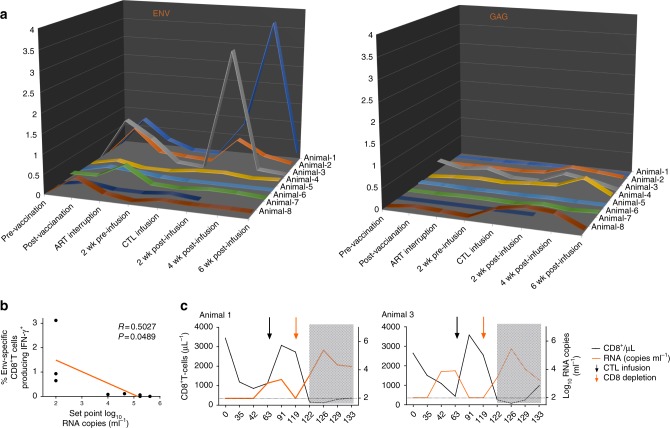
Kinetics of Env-specific immune response and the correlates of virologic control. **a** The frequency of circulating antigen-specific CD8^+^ T-cells in PBMCs was measured at the indicated time points from SHIV controllers (Animals 1–3, *n* = 3), non-controllers (Animals 4–6, *n* = 3) and untreated animals (Animals 7 and 8, *n* = 2). *y*-axis, from Lift to Right, frequency of HIV_CN54_ Env-specific CD8^+^ T-cell producing IFN-γ^+^ and frequency of SIV_mac239_ Gag-specific CD8^+^ T-cell producing IFN-γ^+^. wk, weeks. **b** Correlations between Env-specific CD8^+^ T-cell responses at 2 weeks after CTL infusion and set point log SHIV RNA after CTL infusion (Spearman’s correlation coefficient (R) and P values are indicated. Note that x-axis is log_10_ scales, *n* = 8). **c** Depletion of CD8^+^ T cells in controller macaques results in the rapid induction of plasma viremia. CD8^+^ T-cell count and plasma viral load were shown in SHIV controller macaques following CD8 depletion (Animal 1 and 3, *n* = 2). x-axis, days post- ART interruption. The shaded gray indicated time points post-CD8 depletion

We next evaluated immunologic and virologic correlates of virologic suppression. PBMCs were obtained both pre and post-vaccination, pre and post-infusion of autologous CTLs, and assayed for ICS (Intracellular cytokine staining) response against either Env or Gag peptide pools. We found that therapeutic vaccination with immunogens expressing subtype C-HIV-1_CN54_ Env stimulated antigen-specific CD8^+^ T-cell responses in our model of subtype B/SHIV-SF162P3-infected macaque during ART (Fig. 5a and Supplementary Fig. 2a). The Env-specific CD8^+^T-cells responses were significantly increased compared with pre-vaccination responses (*p* = 0.0067 using Wilcoxon signed-rank test, *n* = 8, Supplementary Fig. 3). However, the vaccine-induced immune responses failed to prevent viral rebound and replication after ART interruption. We found that cellular immune responses gradually declined in the absence of drugs. At 2 weeks before CTL infusion, the T-cell response returned almost to the baseline levels (pre-vaccination). However, the controller macaques (Animal-1, 2 and 3) yielded significantly strong Env-specific CTLs responses at 2 weeks after CTL infusion, and then returned to baseline levels following viremia suppression. Cellular immune responses also correlated directly with virological suppression (Fig. 5b). The frequency of SIV-Gag responses was unchanged. These results strongly supported a cytotoxic mechanism for suppression of SHIV replication.

### In vivo CD8^+^ T-cell depletion

The best evidence for the clinical control of HIV-infected cells by CD8 cytotoxic T cells come from studies relating the numbers and activity of CD8 T cells to viral load^12^. To evaluate whether the observed suppression of SHIV replication was achieved through the elimination of infected CD4^+^T-cells by CTLs, we administered the CD8^+^T-cell-depleting mAb (MT807R1) to the CTL treated controller macaques (Animals 1 and 3). The infusion of the anti-CD8 mAb caused an immediate increase in plasma viral load up to 10^5^ and 10^6^ SHIV RNA copies per ml, commensurate with a decline in CD3^+^ CD8^+^ T cells levels (Fig. 5c). These results suggested that adoptively transferred CTLs were responsible for in vivo of viral levels and that a depletion of this subset could lead to recrudescence of viremia.

## Discussion

An effective strategy for reaching HIV functional cure should elicit immune recognition of sufficient breadth to protect against genetically diverse forms of circulating virus^13^. The induction of a virus-reactive CTL response is therefore critically needed to prevent viral rebound after ART interruption. Here, we propose a therapeutic approach to achieving HIV functional cure by eliciting HIV-specific CTL in vivo using an HIV vaccine to stimulate HIV-specific CTL during ART and, subsequently, amplifying this response ex vivo for adoptive cell therapy as a means of providing long-term suppression of viral rebound following ART interruption.

We demonstrate first that therapeutic vaccination with immunogens expressing subtype C-HIV-1_CN54_ Env could elicit antigen-specific CD8^+^T-cell responses in our model of subtype B/SHIV-SF162P3-infected macaque. Second, we adoptively transferred the therapeutic vaccine induced, ex vivo expanded HIV-specific CTLs following ART interruption and demonstrated viral control, suggestive of cross-reactive viral suppression. Moreover, compared to non-controllers, controller macaques exhibited significantly stronger subtype C-Env-specific CTL responses endowed with central memory properties, poly-functionality, and higher cytotoxicity against Env regions which playing an important role in SHIV control. Although the number of macaques used in this study was limited, our finding of rapid virological suppression by this strategy is encouraging.

In both murine and human studies, CD8^+^ T-cell culture conditions that include IL-21 during in vitro priming have been shown to facilitate greater expansion of the antigen- specific responding T cells in vitro. The effects of IL-21 exposure during priming of antigen-specific CD8^+^ naïve T cells have been well-characterized and shown to imprint a central memory program after antigen-specific stimulation to enhance survival, expansion, and in vivo persistence after infusion^14–17^. By applying this approach to our study, IL-21-primed, Env-specific CD8^+^ T-cells with high replicative capacity were generated from the peripheral blood of each macaque and expanded up to 1000-fold, achieving 1–10 billion cells within 8 weeks for adoptive transfer. Importantly, we noted that the infused products in controller macaques (Animals 1–3) exhibited an early central memory phenotype (CD28^hi^ and CD127^hi^), suggesting a role for these cells in providing durable immunological suppression of the virus in the absence of drugs. However, the effect of IL-21 on previously exposed antigen-experienced memory cells during stimulation is less clear. The precise immunological mechanism(s) mediating virological control warrant further investigation.

It is worth noting that the CTL response elicited by vaccination in the macaques is insufficient, in itself, to completely suppress viral rebound after ART interruption. Long-term viral suppression was observed after CTL therapy only in three of the six macaques who showed low viral rebound or transient viremia suppression after ART interruption. Among the macaques presenting with high viral rebound or without viral control during ART (due to development of drug resistance in this case), infusion of Env-reactive CTL infusion was not able to adequately suppress viral rebound. To achieve more comprehensive control following ART interruption in hopes of HIV functional cure, stronger therapeutic HIV vaccines may need to be developed to generate a more robust CTL response (e.g., by repeated dosing, broader range of targets or selection of higher affinity T cells).

In conclusion, we demonstrate a rapid, consistent and potent strategy to increasing Env- specific CTL responses for adoptive therapy by vaccinating SHIV infected macaques during ART treatment. We have also shown that after adoptive cellular therapy with Env-specific CTL, partial virus suppression can be achieved in macaques with low viral rebound after ART interruption leading to reduction of viremia to undetectable levels for >2 months. Taken together, these results suggest that therapeutic vaccination in combination with adoptive cell therapy warrant further exploration as a strategy for HIV functional cure.

## Methods

### Animals and ethics statement

All animal experiments were reviewed and approved by the Institutional Animal Care and Use Committee (Permit Number: ILAS-VL-2011–001) of the Laboratory Animal Science, Chinese Academy of Medical Sciences and Peking Union Medical College and performed in accordance with the relevant guidelines and regulations. All Chinese-origin rhesus macaques (3–6 years old, 4–6 kg, male or female) were housed at facilities accredited by the Association for Assessment and Accreditation of Laboratory Animal Care (AAALAC) in the Institute of Laboratory Animal Science, Chinese Academy of Medical Sciences and Comparative Medicine Center, Peking Union Medical College, Beijing, China, under the care of licensed veterinarians. Immunologic and virologic assays were performed blinded.

### Drug resistance mutation assay

Viral genome was extracted from plasma samples with the QIAamp Viral RNA Mini Kit (Qiagen, Hilden, Germany). cDNA was reverse transcribed from viral RNA and first-round polymerase chain reaction (PCR) was performed by one-step RT-PCR using primers *POL*-1F(5′-GAAATGCTGACGGCTTGTCAAGGAG-3′) and *POL*-1R (5′-CTCTGCTGCAGGTCCACCATG-3′). The primers of second round PCR were *POL*-2F(5′-tggcagaagccctgaaagaggc-3′) and *POL*-2R(5′-ctatgccacctctctagcctctcc-3′). PCR products were purified with a QIAgen column and sequenced with an ABI 3730 DNA sequencer (Applied Biosystems, Foster City, CA). To determine drug-resistant mutations, sequences were compared with the consensus SIV_mac_ sequence in the HIV2EU Database (http://www.hiv-grade.de/HIV2EU).

### Peptides

20-mers Env and Gag peptide pools (overlapping by 10 amino acids) from HIV-1_CN54_ strain (Bio-scientific Co., Shanghai, China) and SIV_Mac239_ strain (NIH AIDS Research and Reference Reagent Program, USA) were used in this study.

### Isolation of rhesus macaque PBMCs

Rhesus macaque PBMCs were isolated from EDTA-treated venous blood by Ficoll Hypaque centrifugation (Sigma) within 6 h of collection. After cell counting, PBMCs were resuspended in R10 medium (RPMI-1640 medium supplemented with 10% FBS, 2 mmol L-glutamine, 25 mM HEPES, 100 U/ml penicillin, 100 mg/ml streptomycin) for next experiments^18^.

### Flow cytometry

PBMCs isolated from each macaque were stimulated with Env and Gag peptide pools (2 μg/ml) in the presence of Brefeldin A (Sigma, Catalogue# B-7651), monensin (GolgiStop, BD Biosciences, Catalogue# 554724), and CD107a (H4A3, BD Biosciences, Catalogue# 561348) for 6 h. Dimethyl sulfoxide (DMSO, Sigma, Catalogue# D-5879) was used as negative control for peptides. As a positive control, cells were stimulated with a combination of PMA and ionomycin. After stimulation, cells were washed with phosphate-buffered saline (PBS, Corning) and stained with an ultraviolet-excitable, amine-reactive viability dye (LIVE/DEAD, Invitrogen, Catalogue# L-23105) to exclude dead cells. Cells were subsequently washed with staining buffer (PBS containing 2% fetal calf serum and 1 mM EDTA) and stained with anti-CD3 (SP34.2, dilution 1/400), anti-CD8 (SK1, dilution 1/50), anti-CD4 (L200, dilution 1/100), anti-CD95 (DX2, dilution 1/100), anti-CD28(CD28.2, dilution 1/50), anti-IFN-γ (B27, dilution 1/50), anti-TNF-α (Mab11, dilution 1/50), anti-MIP-1 β (D21-1351, dilution 1/100), anti-CCR5 (2D7, dilution 1/100), anti-HLA-DR (LN3, dilution 1/200), PE Mouse Anti-Ki67 Set (Catalogue# 556027) and anti-CD69 (FN50, dilution 1/100) from BD Biosciences, with anti-CD127 (A019D5, dilution 1/100) and anti-CCR7 (G043H7, dilution 1/50) from BioLegend, and with Anti-PD-1 (eBioJ105, dilution 1/100) from eBioscience. Initial gating was performed with a forward scatter (area) versus side scatter (area) lymphocyte gate. CD4 and CD8 cells consisted of live (viability stain negative) CD3^+^T cells^18^. In Fig. 3a–c, following initial gating and identification of CD8^+^ T cells, a gate was made for each respective function using combinations that provided optimal separation (Supplementary Figure 2a). After the gates for each function were created, the full array of possible combinations was created, equating to 16 response patterns when testing 4 functions. Data are reported after background correction. The cutoff for a positive response is dependent on the particular combination from 3 and 4 functions to 1 function^19,20^. Flow cytometry data were acquired using a Fortessa LSR flow cytometer (LSRFortessa^TM^, BD), equipped with FacsDiva^TM^ software (BD). Data analysis was performed using FlowJo^TM^ software (Tree Star).

### Stimulation of Env-specific CD8^+^ T cells

Macaque PBMCs were stimulated twice for 7d with autologous antigen presenting cells (APCs). *γ*-Irradiated autologous PBMCs were used as APCs^21^. Cells were irradiated in a *γ-irradiator* (Beijing Normal University, Beijing, China) at 10,000 rad. APCs were pulsed for 2 h with Env peptide pools (2 μg/ml) in PBS, washed, and then co-cultured in 48 well plates with CD4 depleted PBMC in R10 medium. The stimulations were supplemented with IL-21 (30 ng/ml, PeproTech, Catalogue# 200–21) on day 1. On day 2 of each stimulation, the IL-2 (12.5 IU/ml, PeproTech, Catalogue# 200–02), IL-7 (5 ng/ml, PeproTech, Catalogue# 200–07), and IL-15 (1 ng/ml, PeproTech, Catalogue# 200–15) were added.

### Isolation and expansion of CTL-lines

Following two rounds of peptide stimulation, the Env-specific CD8^+^ T cells were isolated by non-human primate CD8^+^ T-cell isolation kit (Miltenyi Biotec, Catalogue# 130-092-143) and expanded using the Rapid Expansion Protocol^22^ in a sterile 25-cm^2^ tissue culture flasks. The CD8^+^ T-cell lines were expanded in vitro for 5–7 weeks through repeated cycles of bi-weekly stimulation with CD3 monoclonal antibody (30 ng/mL, SP34-2, BD Biosciences, Catalogue# 551916) and *γ*-irradiated allogeneic PBMCs and EBV-transformed B cell lines (TM B-LCL) as feeder cells to obtain billions of cells of each macaques. Cultures were add on the first, fourth, seventh and tenth days with IL-2 (50 IU/ml, PeproTech, Catalogue# 200–02) and were split to maintain a density of less than 2 × 10^6^ cells/ml. To expand cells for therapy, the cells were stimulated in multiple 75-cm^2^ tissue culture flasks with anti-CD3 monoclonal antibody and feeder cells, and were add on the first, fourth, seventh and tenth days of a 14-day stimulation cycle. The feeder cells were irradiated in a *γ-irradiator* (Beijing Normal University, Beijing, China) at 10000 rad (PBMCs) or 12,500 rad (TM B-LCL).

Upon infusion, the required number of expanded CD8^+^T-cells was resuspended at 1 × 10^9^ cells in 10 ml sterile PBS and infused intravenously into autologous macaques. To support survival of infused T cells, animals were given low-dose (10^4^ IU/ kg) IL-2 (PeproTech, Catalogue# 200–02) injections following transfers.

### Phenotypic and functional characterization of CTL-lines

In vitro specific antigen recognition of CTL-lines was determined by flow cytometry following stimulation with *γ*-Irradiated autologous PBMCs pulsed with Env peptide pools (2 μg/ml). *γ*-irradiated autologous PBMCs were used as APCs^21^. Non-pulsed autologous PBMCs were included as negative control. CD107a (H4A3, BD Biosciences, Catalogue# 561348) was added to the cell suspensions before incubation. The cultures were incubated for 6 h at 37 ℃ in a 5% CO_2_ incubator, followed by in the presence of the secretion inhibitor monensin (GolgiStop, BD Biosciences, Catalogue# 554724) and Brefeldin A (Sigma, Catalogue# B-7651). 10^6^ Cells were washed and then stained with anti-CD3 (SP34.2, dilution 1/400), anti-CD4 (L200, dilution 1/100), anti-CD8 (SK1, dilution 1/50) and anti-CD95 (DX2, dilution 1/100) from BD Biosciences at room temperature for 20 min. Dead cells were excluded by using LIVE/DEAD Fixable Dead Cell Stain Kit (Invitrogen, Catalogue# L-23105). Cells were permeabilized using Invitrogen Fixation Medium A (Catalogue# GAS001S100) for 30 min at 4 °C, and stained with antibodies for anti-TNF-α (Mab11, dilution 1/50), anti-MIP-1β (D21-1351, dilution 1/100) and anti-IFN-γ (B27, dilution 1/50) in Invitrogen Permeabilization Medium B (Catalogue# GAS002S100) for 30 min at 4 °C.

In vitro early central memory (CD28^+^ and CD127^+^) phenotypic characterization of CTL-lines was determined following stimulation with SHIV-SF162P3-infected autologous CD4^+^ T cells. Briefly, CTL-lines were cocultured with autologous infected CD4^+^ T cells for 6 h. Cells were washed and analyzed for surface expression of CD28, CD95 and CD127. Uninfected autologous CD4^+^ T cells were included as negative control. All data were acquired using a Fortessa LSR flow cytometer (LSRFortessa^TM^, BD), equipped with FacsDiva^TM^ software (BD). Data analysis was performed using FlowJo^TM^ software (Tree Star).

### Virus inhibition assay

CD4^+^ T-cells were isolated from PBMCs by positive selection (Miltenyi Biotec, Catalogue# 130-092-144) and activated with 5 μg/ml Phytohemagglutinin-L (PHA-L, Sigma, Catalogue# L2769) and 100 IU/ml IL-2 (PeproTech, Catalogue# 200–02) for three days and then infected with SHIV-SF162P3 at a multiplicity of infection of 0.01^18^. After 4 h of incubation with the virus, infected cells were washed twice and resuspended at R10 medium with IL-2 (50 IU/ml) and were cultured in triplicate in 96-well plates at 10^5^ cells/well, alone (positive control) or together with autologous CTL-lines at an effector-to- target ratio of 1:1, 2:1 and 5:1. Culture medium was changed at day 4, 7 and 10. The level of p27 antigen in the supernatant at day14 was determined by enzyme-linked immunosorbent assay (ELISA, Express Biotech, Catalogue# SK845). SHIV-suppressive capacity of CD8^+^T cells (Log p27 decrease)^23^ =

$${\mathrm{Log}}_{{\mathrm{10}}}\frac{{\hbox{mean p27 pg/ml in cultures of SHIV infected in vitro}}{-}{\hbox{targets at peak of viral replication}}}{{\hbox{mean p27 pg/ml in infected in vitro}}{-}{\hbox{targets: CD8 co}}-{\hbox{cultures at the same time point}}}$$

### Plasma viral load and T-cell count

SHIV-SF162P3 plasma viral load were assessed with real-time RT-PCR using primers and probe for the *gag* sequence (TaqMan EZ RT-PCR Core Reagent Kit; ABI, Waltham, USA). All specimens were extracted and amplified in duplicate, and the mean results were reported. With an input of 0.2 ml plasma, the assay had a sensitivity of 100 viral RNA copies per ml of plasma. CD4^+^ and CD8^+^ T-cell counts from whole blood were determined by an International External Quality Assessment (UK NEQAS)-certified laboratory using FACS Calibur^TM^ (BD).

### In vivo CD8^+^ T-cell depletion

Macaques were injected subcutaneously with anti-CD8 mAbα M-T807R1 (NIH Nonhuman Primate Reagent Resource, USA) (10 mg/kg) on day 0, and intravenously (5 mg/ kg) on days 3, 7, and 10.

### Statistical analysis

Flow cytometry data were analyzed with FlowJo^TM^ software (Tree Star,). Statistical analyses were performed using GraphPad Prism (GraphPad Software). Statistical analyses involved Wilcoxon matched pairs signed-rank tests for paired analyses, and Spearman rank-correlation tests for correlation analyses. Comparisons between groups were made by Student’s t-test. All tests were 2-tailed, and *P* < 0.05 was accepted as statistically significant.

### Reporting Summary

Further information on experimental design is available in the Nature Research Reporting Summary linked to this article.

## Supplementary information

Supplementary Information

Reporting Summary

## Data Availability

Sequence data that support the findings of this study have been deposited in the international Genbank http://www.hiv.lanl.gov/components/sequence/HIV/search/search.html with the accession numbers of MK638909 to MK638920. All other data are available from the corresponding author upon request.
