# Supplementary material for: CTL-mediated immunotherapy can suppress SHIV rebound in ART-free macaques
Source: Nat Commun. 2019 May 21;10:2257. doi: 10.1038/s41467-019-09725-6 (PMC6529452; doi:10.1038/s41467-019-09725-6)
Supplement: Supplementary file 1 — Supplementary Information [file 41467_2019_9725_MOESM1_ESM.pdf]

CTL-mediated immunotherapy can suppress SHIV rebound in ART-free macaques

Fan et al.

Supplementary Figures

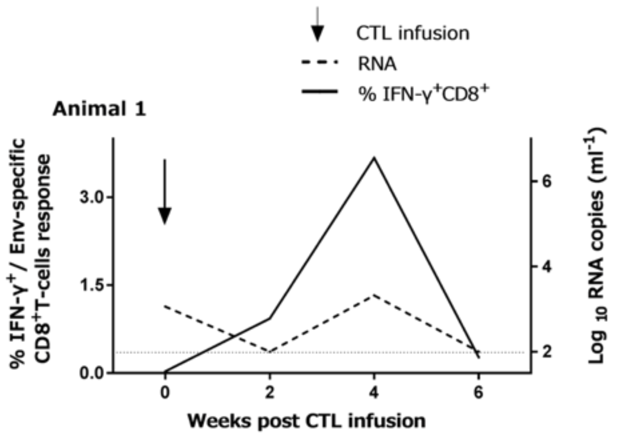

Supplementary Figure 1 | Kinetics of viremia control and CD8$^+$ T cell response. Kinetics of viremia control in Animal 1 treated by expanded Env-specific CD8$^+$T-cells. Limit of detection, 2 Log RNA copies per ml.

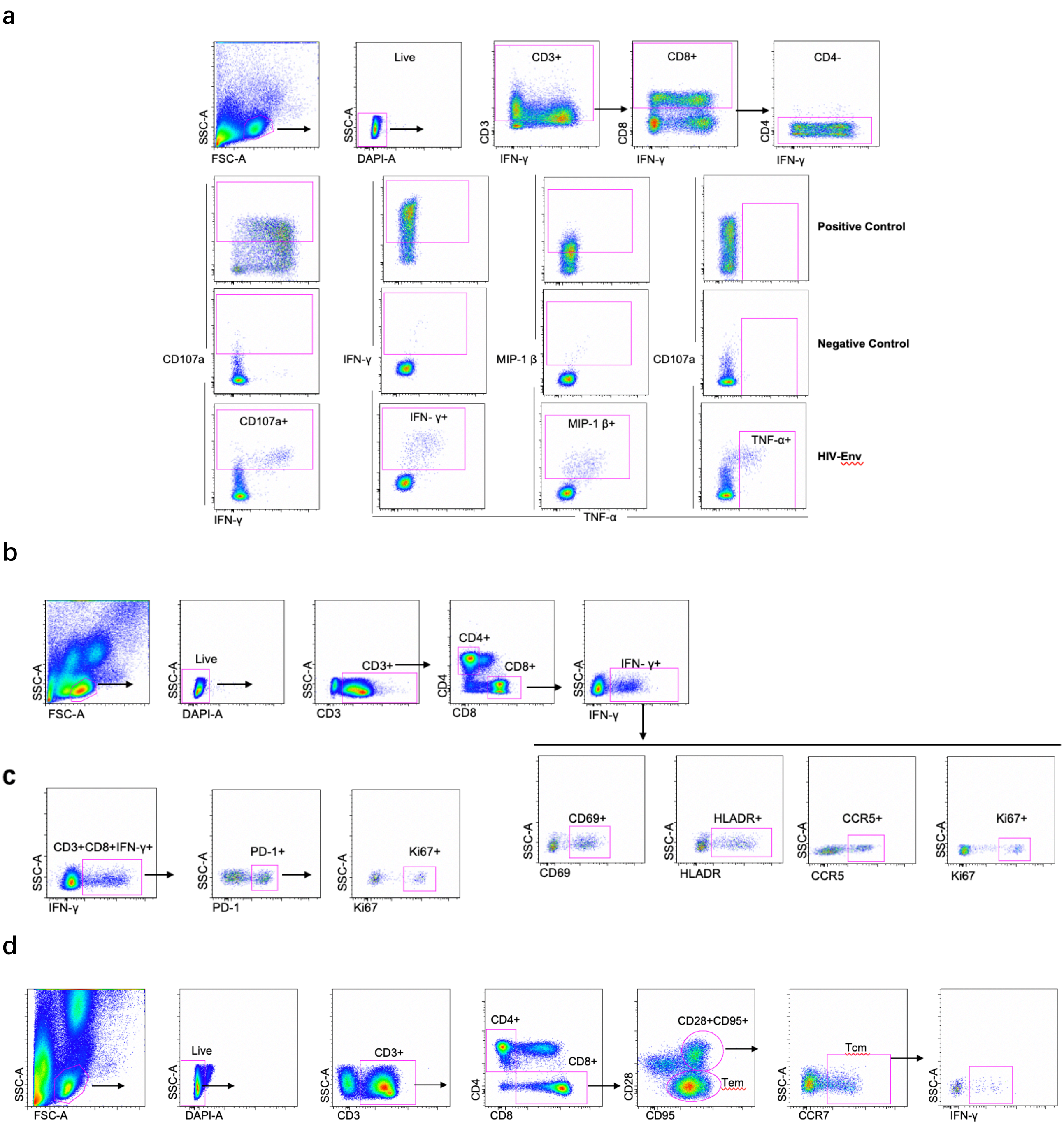
a
SSC-A
FSC-A
Live
DAPI-A
CD3+
CD3
IFN-γ
CD8+
CD8
CD4-
CD4
Positive Control
Negative Control
HIV-Env
CD107a
IFN-γ
MIP-1 β
CD107a+
IFN- γ+
MIP-1 β+
TNF-α+
TNF-α
b
CD4+
CD8+
IFN- γ+
c
CD3+CD8+IFN-γ+
PD-1+
PD-1
Ki67+
Ki67
CD69+
CD69
HLADR+
HLADR
CCR5+
CCR5
d
CD28+CD95+
CD28
CD95
Tem
Tcm
CCR7

e

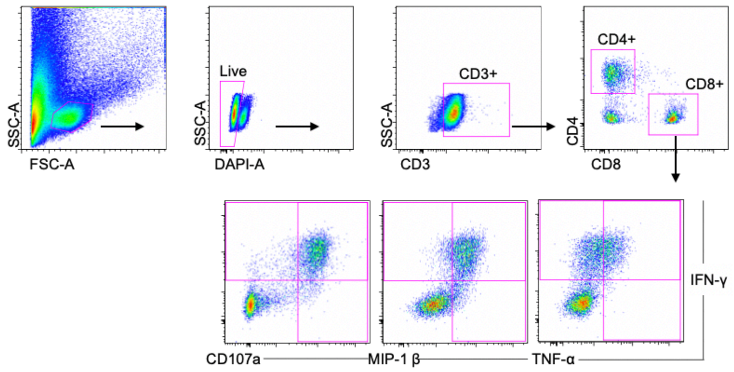

f

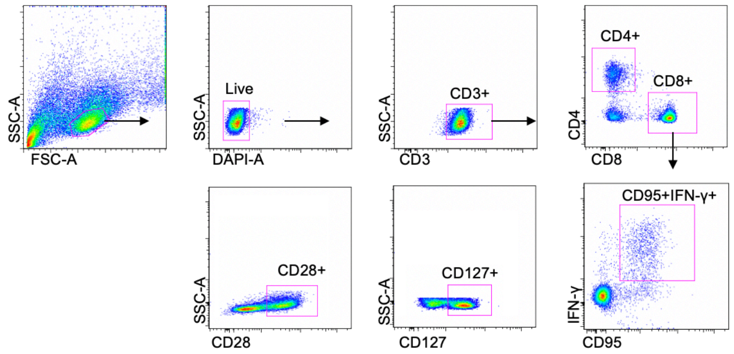

Supplementary Figure 2 | Gating strategies used for this study. (**a**) Gating strategy for identification of multifunctional $CD8^{+}$T-cell responses (Figure 3a, c and Figure 5a). (**b**) Gating strategy to analysis activation profile of pre- and post CTL infusion presented on Figure 3b. (**c**) Gating strategy to analysis expression of PD-1^{+}Ki67^{+} on Env-specific $CD8^{+}$ T-cells pre- and post-CTL infusion presented on Figure 3e. (**d**) Gating strategy for central memory T cells pre-and post-CTL infusion analysis (Figure 3d). (**e**) Gating strategy for identification of phenotypic and functional characterization of CTL-lines (Figure 4a). (**f**) Gating strategy for memory phenotypic characterization of CTL-lines presented on Figure 4e.

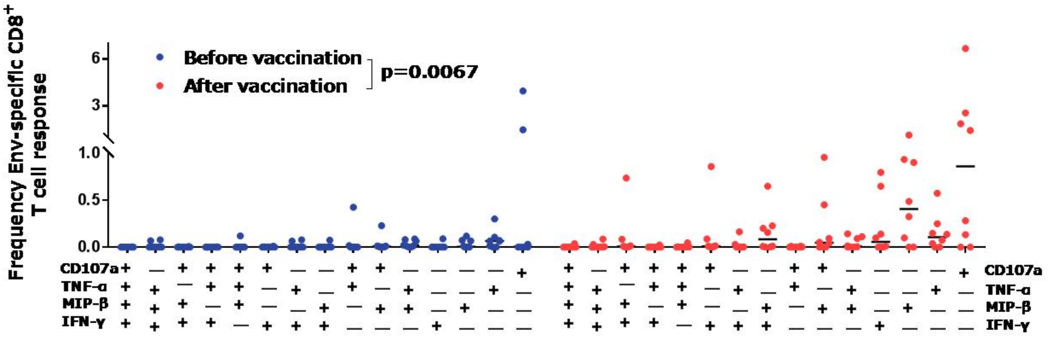

Supplementary Figure 3 | Functional profiles of CD8$^+$T-cells pre- and post-vaccination. Functional profiles of PBMCs CD8$^+$T-cells pre- and post-vaccination in response to 6-hour incubation with 2μg/mL peptide pools, displaying as the frequencies of Env-specific CD8$^+$T-cells producing CD107a, TNF-α, MIP-1β and IFN-γ ($p=0.0067$ using Wilcoxon signed-rank test, $n = 8$).

Supplementary Tables

Supplementary Table 1 | Genotyping result to SHIV drug resistance mutations.

Animal Number	Baseline	ART 3Weeks	ART 16Weeks	ART 38Weeks
Animal-7	No DR			No DR
Animal-8	No DR			No DR
Animal-5	No DR	M184V*	M184V	M184V
Animal-6	No DR	M184V	M184V	M184V

*Drug resistance mutation to Lamivudine (3TC)

Supplementary Table 2 | SHIV-infected CD4$^+$ T-cell elicit CD107a expression in CTL-lines. CTL-lines were cocultured with autologous infected CD4$^+$ T cells for 6h. The frequency of CD107a expressing CTL was determined by flow cytometry. CTL exposed to uninfected CD4$^+$ T cells were used to estimate background levels of CD107a.

	Background level (%)	Cytotoxicity of CTL-lines (%)
Animal-1	3.3	96.6
Animal-2	4.92	86.3
Animal-3	2.59	95.2
Animal-4	4.44	88.3
Animal-5	5.06	93.9
Animal-6	6.98	84.4
