# Supplementary material for: CTL-mediated immunotherapy can suppress SHIV rebound in ART-free macaques
Source: Nat Commun. 2019 May 21;10:2257. doi: 10.1038/s41467-019-09725-6 (PMC6529452; doi:10.1038/s41467-019-09725-6)
Supplement: Supplementary file 2 — Reporting Summary [file 41467_2019_9725_MOESM2_ESM.pdf]

nature research

Corresponding author(s): Yiming Shao & Cassian Yee

Last updated by author(s): Mar 11, 2019

Reporting Summary

Nature Research wishes to improve the reproducibility of the work that we publish. This form provides structure for consistency and transparency in reporting. For further information on Nature Research policies, see Authors & Referees and the Editorial Policy Checklist.

Statistics

For all statistical analyses, confirm that the following items are present in the figure legend, table legend, main text, or Methods section.

n/a	Confirmed	
☐	☒	The exact sample size (*n*) for each experimental group/condition, given as a discrete number and unit of measurement
☐	☒	A statement on whether measurements were taken from distinct samples or whether the same sample was measured repeatedly
☐	☒	The statistical test(s) used AND whether they are one- or two-sided<br>*Only common tests should be described solely by name; describe more complex techniques in the Methods section.*
☐	☒	A description of all covariates tested
☐	☒	A description of any assumptions or corrections, such as tests of normality and adjustment for multiple comparisons
☐	☒	A full description of the statistical parameters including central tendency (e.g. means) or other basic estimates (e.g. regression coefficient) AND variation (e.g. standard deviation) or associated estimates of uncertainty (e.g. confidence intervals)
☐	☒	For null hypothesis testing, the test statistic (e.g. *F*, *t*, *r*) with confidence intervals, effect sizes, degrees of freedom and *P* value noted<br>*Give P values as exact values whenever suitable.*
☐	☒	For Bayesian analysis, information on the choice of priors and Markov chain Monte Carlo settings
☐	☒	For hierarchical and complex designs, identification of the appropriate level for tests and full reporting of outcomes
☐	☒	Estimates of effect sizes (e.g. Cohen's *d*, Pearson's *r*), indicating how they were calculated

Our web collection on statistics for biologists contains articles on many of the points above.

Software and code

Policy information about availability of computer code

Data collection	No software was used.
Data analysis	No software was used.

For manuscripts utilizing custom algorithms or software that are central to the research but not yet described in published literature, software must be made available to editors/reviewers. We strongly encourage code deposition in a community repository (e.g. GitHub). See the Nature Research guidelines for submitting code & software for further information.

Data

Policy information about availability of data

All manuscripts must include a data availability statement. This statement should provide the following information, where applicable:

- Accession codes, unique identifiers, or web links for publicly available datasets
- A list of figures that have associated raw data
- A description of any restrictions on data availability

Sequence data that support the findings of this study have been deposited in the international gene sequence bank (seq-info@lanl.gov/Job#9910-GB9910). All other relevant data are available from the corresponding author upon reasonable request.

Field-specific reporting

Please select the one below that is the best fit for your research. If you are not sure, read the appropriate sections before making your selection.

☒ Life sciences ☐ Behavioural & social sciences ☐ Ecological, evolutionary & environmental sciences

October 2018

Life sciences study design

All studies must disclose on these points even when the disclosure is negative.

Sample size	8 Rhesus macaque were used in this study. The PBMCs were isolated from EDTA-treated venous blood by Ficoll Hypaque centrifugation (Sigma) within 6 h of collection. Determined cell number and viability according to Guava Counter. Statistical analyses involved Wilcoxon matched pairs signed-rank tests for paired analyses, and Spearman rank-correlation tests for correlation analyses. Comparisons between groups were made by Student's t-test. All tests were 2-tailed, and $P < 0.05$ was accepted as statistically significant.
Data exclusions	No data were excluded from the analyses.
Replication	All plasma specimens were extracted and amplified in duplicate, and the mean results were reported. Flow cytometry data were not replicated since the number of macaque PBMCs samples collected is limited.
Randomization	8 Chinese-origin Rhesus macaques were infected with the simian-human immunodeficiency virus SHIV-SF162P3(3×106 TCID50) by intrarectal route and treated with ART regimens initiating from week 8. After ART interruption, autologous CTL infusion were given upon viral rebound macaques. All allocation was random.
Blinding	Immunologic and virologic assays were performed blinded.

Reporting for specific materials, systems and methods

We require information from authors about some types of materials, experimental systems and methods used in many studies. Here, indicate whether each material, system or method listed is relevant to your study. If you are not sure if a list item applies to your research, read the appropriate section before selecting a response.

Materials & experimental systems

n/a	Involved in the study	
☒	☐	Antibodies
☒	☐	Eukaryotic cell lines
☒	☐	Palaeontology
☐	☒	Animals and other organisms
☒	☐	Human research participants
☒	☐	Clinical data

Methods

n/a	Involved in the study	
☒	☐	ChIP-seq
☐	☒	Flow cytometry
☒	☐	MRI-based neuroimaging

Animals and other organisms

Policy information about studies involving animals; ARRIVE guidelines recommended for reporting animal research

Laboratory animals	Chinese-origin rhesus macaques, 3-6 years old, 4-6kg, male or female, under the care of licensed veterinarians.
Wild animals	The study did not involve wild animals.
Field-collected samples	All rhesus macaques were housed in facilities accredited by the Association for Assessment and Accreditation of Laboratory Animal Care (AAALAC) in the Institute of Laboratory Animal Science, Chinese Academy of Medical Sciences. The animals were monitored continuously by veterinarians to ensure their welfare and anesthetized with ketamine-HCl (10mg/kg) prior to all traumatic procedures. The anesthetics were used to minimize pain and distress associated with the experimental procedures, in accordance with the recommendations of the Weatherall Report.
Ethics oversight	All animal experiments were reviewed and approved by the Institutional Animal Care and Use Committee (Permit Number: ILAS-VL-2011-001) of the Laboratory Animal Science, Chinese Academy of Medical Sciences and Peking Union Medical College and performed in accordance with the relevant guidelines and regulations.

Note that full information on the approval of the study protocol must also be provided in the manuscript.

Flow Cytometry

Plots

Confirm that:

☒ The axis labels state the marker and fluorochrome used (e.g. CD4-FITC).

☒ The axis scales are clearly visible. Include numbers along axes only for bottom left plot of group (a 'group' is an analysis of identical markers).

☒ All plots are contour plots with outliers or pseudocolor plots.

☒ A numerical value for number of cells or percentage (with statistics) is provided.

Methodology

Sample preparation	Cryopreserved macaque peripheral blood mononuclear cells (PBMCs) were thawed and rested in R10 for 2 hours prior to use.
Instrument	All data were collected on BD LSRFortessa instrument (BD Biosciences) .
Software	Flow cytometry data were equipped with FacsDivaTMsoftware (BD, USA). Data analysis was performed using FlowJoTMsoftware (Tree Star, USA).
Cell population abundance	The number of CD8+T cells, as counted in the CD8 gates determined by the software analysis of the data after collection is less than 10,000 for any of the wells for a particular sample.
Gating strategy	Initial gating was performed with a forward scatter (area) versus side scatter (area) lymphocyte gate. CD4 and CD8 cells consisted of live (viability stain negative) CD3+T cells. All response data shown have been background- subtracted on the basis of the unstimulated control for each sample set. We provided graphically account for all FACS sequential gating in Supplementary Figures.

☒ Tick this box to confirm that a figure exemplifying the gating strategy is provided in the Supplementary Information.
